# Combinations of Kinase Inhibitors Protecting Myoblasts against Hypoxia

**DOI:** 10.1371/journal.pone.0126718

**Published:** 2015-06-04

**Authors:** Yunyi Kang, Matthew Tierney, Edison Ong, Linda Zhang, Carlo Piermarocchi, Alessandra Sacco, Giovanni Paternostro

**Affiliations:** 1 Sanford-Burnham Medical Research Institute, La Jolla, California, United States of America; 2 Salgomed Inc, Del Mar, California, United States of America; 3 Department of Physics and Astronomy, Michigan State University, East Lansing, Michigan, United States of America; Università degli Studi di Firenze, ITALY

## Abstract

Cell-based therapies to treat skeletal muscle disease are limited by the poor survival of donor myoblasts, due in part to acute hypoxic stress. After confirming that the microenvironment of transplanted myoblasts is hypoxic, we screened a kinase inhibitor library *in vitro* and identified five kinase inhibitors that protected myoblasts from cell death or growth arrest in hypoxic conditions. A systematic, combinatorial study of these compounds further improved myoblast viability, showing both synergistic and additive effects. Pathway and target analysis revealed CDK5, CDK2, CDC2, WEE1, and GSK3β as the main target kinases. In particular, CDK5 was the center of the target kinase network. Using our recently developed statistical method based on elastic net regression we computationally validated the key role of CDK5 in cell protection against hypoxia. This method provided a list of potential kinase targets with a quantitative measure of their optimal amount of relative inhibition. A modified version of the method was also able to predict the effect of combinations using single-drug response data. This work is the first step towards a broadly applicable system-level strategy for the pharmacology of hypoxic damage.

## Introduction

Skeletal muscle wasting is characterized by a progressive loss of muscle mass and function, compromising patient quality of life and survival [1]. Muscle degeneration is accompanied by a progressive exhaustion of muscle stem cell function, essential for tissue homeostasis and repair [2]. Cell replacement therapies have been the subject of intense studies in the recent years in order to restore regenerative potential [3–7]. However, a major limitation of cell-based strategies is the poor survival of transplanted cells within skeletal muscles [8–10]. *In vivo*, most transplanted myoblasts die in the first few days following transplantation, preventing their participation to tissue regeneration. This is at least in part due to the hypoxic environment, as large number of cells transplanted into a solid organ form a mass in which blood vessels are not present, hence limiting the oxygen supply [11–13].

Hypoxia activates a complex set of pathways, supporting the development of a system-level therapeutic approach. Although cellular hypoxia promotes cell death, the lack of oxygen supply also activates several adaptive pathways to promote survival. These include a switch to anaerobic metabolism by enhancing glycolysis and inhibiting the Krebs cycle, a switch from anabolic to catabolic pathways to limit energy expenditures and the activation of autophagy, a key adaptive response to cellular stress [14, 15]. Approaches targeting angiogenesis and stress proteins have been reported to improve myoblast survival upon transplantation. These factors include Hypoxia Inducible Factor 1 alpha (HIF1α), Vascular Endothelial Growth Factor (VEGF), and Heat Shock Proteins [11, 16–19]. The identification of drugs that can confer hypoxia resistance would improve the outcome of myoblast replacement therapy, possibly in combination with these approaches. Protein kinases are the key regulators of numerous cellular signaling pathways and multiple kinase pathways are involved in the responses to hypoxic stresses. Thus, simultaneously targeting several kinases involved in the hypoxia-induced cellular death processes might help to protect myoblasts from hypoxia.

In this study, we screened for kinase inhibitors that affect hypoxia-resistance *in vitro*. Several candidate kinase inhibitors were identified with potent effects on primary myoblast survival under hypoxia. Fully factorial analysis uncovered kinase inhibitor combinations able to both additively and synergistically improve myoblast survival. Using a pathway analysis and a novel statistical method developed by our group [20], we have identified key kinases influencing hypoxia-induced signaling in myoblasts. The method was modified to allow for predictions on combinations containing up to four drugs, which were validated experimentally. The modified method assumes a specific dependence (defined by Eq 1, 2 and 3) of cell viability as a function of profiling parameters of drugs used in a combination. Collectively, the experimental results and the updated statistical analysis proposed in this study establish a methodology for identifying drugs and drug combinations promoting myoblast survival under hypoxic conditions. This approach might further the transition towards cell-based therapeutic application for the treatment of skeletal muscle degenerative diseases.

## Materials and Methods

### Animals

All protocols were approved by the Sanford-Burnham Medical Research Institute Animal Care and Use Committee. C57BL/6, *NOD/SCID* and *EGFP* mice were purchased from Jackson Laboratories. *Luciferase* mice [21] were kindly provided by H. M. Blau (Stanford University) and crossed with *EGFP* mice to generate *Luciferase* x *EGFP* mice. All mice used for transplantation experiments were 2–3 months of age. Local hind limb irradiation was performed following ketamine-xylazine administration (75 and 5 mg/kg). Intramuscular transplantation and non-invasive bioluminescence imaging was performed under 1–4% 1L O_2_/min isoflurane inhalation. Euthanasia was performed under isoflurane inhalation followed by cervical dislocation.

### Cell culture

Primary myoblasts were isolated from skeletal muscle of 2 month old C57BL/6 and *Luciferase* x *EGFP* mice as described previously [22], plated on tissue culture plates coated with collagen (BD Biosciences) and maintained in growth media (45% DMEM, 40% F10, 15% FBS and 2.5 ng ml^-1^ bFGF). To expose cells to normoxic (20% O_2_) or hypoxic (~1% O_2_) culture conditions, cultures were placed in an airtight modular hypoxia chamber adjusted to the indicated oxygen concentration.

### Kinase inhibitor library screens

The EMD kinase inhibitor library was screened for their capability to protect cells from hypoxia-induced myoblast cell death/growth arrest. The cells were plated at 1500 cells/well in 384-well plates in growth media. At least 4 hours after cell seeding, 244 kinase inhibitors were dispensed into the cells-seeded plates at 1 μM final concentration using Echo liquid handler (Labcyte). The cells were cultured under hypoxic environment created by the infusion of a gas mixture of 95% of N_2_ and 5% of CO_2_ into an airtight modular hypoxia chamber for 5 days. Two independent screens were performed with duplicates each run.

### Cell survival assay

Relative cell survival was measured by the luciferase-based assay, ATPlite (PerkinElmer), which measures ATP levels in the metabolically active cells according to the manufacturer’s protocol. Luminescence was read with Analyst HT apparatus (Molecular Devices). The counting of viable cells was performed based on brightfield imaging and dye exclusion following the addition of 0.4% Trypan Blue (Invitrogen) and automated cell counting using the Celigo Imaging Cytometer (Nexcelom Bioscience).

### Fully factorial studies

Fully factorial kinase inhibitor combinations were generated in CSV file format using our lab-developed high throughput screening (HTS) manager software. The input files that contain combinations were imported into the Echo Cherry Pick software and kinase inhibitor droplets were transferred from the source plates to the destination plates in 2.5 nl increments accordingly.

### Immunohistochemistry

Muscle tissues were prepared for histology as previously described [23]. Cells and muscle sections were fixed with 1.5% PFA, permeabilised in 0.3% Triton and blocked in 20% goat serum. Incubation with the primary antibodies was performed overnight at 4°C. The antibodies used are: rabbit anti-GFP (Invitrogen), rat anti-laminin (Millipore), rabbit anti-hypoxyprobe (Chemicon), rabbit anti-HIF-1α (Novus Biologicals), rabbit anti-cCasp3 (BD Biosciences) and Alexa-conjugated secondary antibodies (Invitrogen). Images of cell cultures as well as muscle transverse sections were acquired using an inverted epifluorescent microscope (Nikon TE300), 10x objective lens, CCD SPOT RT camera and SPOT imaging software (Diagnostic Instruments). Fluorescent intensities of selected immunofluorescent regions were measured as mean gray values (ImageJ). All images were composed, edited and modifications applied to the whole image using Photoshop CS6 (Adobe).

### Pathway analysis

Pathway analysis was obtained by combining two datasets containing drug-target information[24, 25], one datasets containing protein-protein interactions[26], and a dataset with transcriptional regulation information.[27] Cutoffs corresponding to an IC_50_ of less than 2000 nM and 5% residual kinase activity have been used to indicate a significant action of an inhibitor to a given target.

### Synergy analysis

We have used three different methods to quantify synergy: excess with respect to the Bliss independence model, excess with respect to the highest single agent (HSA) and excess with respect to the highest pair agents (HPA). In contrast to the widely-used Loewe synergy[28], these measures of synergy are well defined for combinations with more than two drugs, and do not require *ad hoc* measurements. The Bliss model[29] assumes that drugs in a combination act independently leading to a cell survival probability that is the product of the survival probabilities under each drug given separately. Deviations from this product model indicate that the drug actions are not independent and quantify synergy if positive or antagonism if negative. The Bliss synergy then defined as

$$S_{Bliss}=v(d_{1},d_{2},d_{3},d_{4},d_{5})-v_{1}(d_{1})*v_{2}(d_{2})*v_{3}(d_{3})*v_{4}(d_{4})*v_{5}(d_{5}),$$

where *v* is the cell viability measured using the combination, *d*_*i*_ is the dose of drug *i*, and *v*_*i*_(*d*_*i*_) is the viability measured using only using drug *i*. The HSA model assumes that if the effects of a combination exceeds those of its components, then they are necessarily helping each other. The synergy can then be quantified looking at deviations from the best of the single agent viabilities at the corresponding doses:

$$S_{HSA}=v(d_{1},d_{2},d_{3},d_{4},d_{5})-Max[v_{1}(d_{1}),v_{2}(d_{2}),v_{3}(d_{3}),v_{4}(d_{4}),v_{5}(d_{5})].$$

Note that in this case the expected action of the drugs is to increase cell survival. Since we are dealing with combinations with up to 5 drugs, we have generalised the HSA synergy measure to take into account deviations from the best pair of drugs. This measure indicates how important is to include more than two drugs in the combinations. This synergy is the Highest Pair Agent (HPA), and we defined it as

$$S_{HPA}=v(d_{1},d_{2},d_{3},d_{4},d_{5})-Max_{i,j}[v_{i}(d_{i}),v_{ij}(d_{i},d_{j})],$$

where *v*_*ij*_(*d*_*i*_, *d*_*j*_) is the viability measured using only the pair of drugs *i* and *j* at the doses *d*_*i*_, *d*_*j*_.

### A regression model

We used the Kinase Inhibitors Elastic Net (KIEN) method[30] to identify the kinase targets more likely to be responsible for the protective effects of the kinase inhibitors. We were also able to predict the effects of combinations four kinase inhibitors (G13, O20, I15, and K10) using a modification of the original KIEN method. These kinase inhibitors were selected for their ability to protect primary myoblasts against hypoxia. Out of 244 drugs from the EMD library, we first selected 58 drugs that have a positive effect on cell survivability against hypoxia. Single-drug screening results with these drugs were used as a training set to build a regression model that uses the kinase catalytic activity as predictor of the viability. The modified KIEN method assumes a dependence of the viability *v*_*i*_ of the form

$$v_{i}=e^{\beta_{0}}\;*{\left(A_{1,i}\right)}^{\beta_{1}}\;*\ldots *{\left(A_{p,i}\right)}^{\beta_{p}},$$ (1)

where we indicate as *A*_*k*,*i*_ the residual activity of the kinase *k* under the effect of drug *i*. The residual activities were obtained from a published dataset containing catalytic activities of kinase inhibitor targets.[24] P17 was also found to protect primary myoblasts, but was excluded in our analysis since the kinase profiling information was not available in this dataset[24].

Eq (1) can be reduced to a linear form using a logarithmic transformation, and the coefficients (*β*_0_, *β*_1_,…, *β*_*p*_) can obtained using a linear regression procedure. The resulting coefficients *β*_*k*_ can be interpreted as a measure of the protective impact of kinase *k* on the cell viability against hypoxia. A higher value of |*β*_*k*_| indicates a higher protective effect of that kinase on the survivability.

The coefficients *β*_*k*_ were then used to predict the viability of four-drug combinations. We assumed that the residual activity *A*_*k*,*c*_ of the kinase *k* under the effect of a four-drug combination *c* with dosage *d* = (*d*_1_, *d*_2_, *d*_3_, *d*_4_) is

$$A_{k,c}=A_{k,1}^{d_{1}}*A_{k,2}^{d_{2}}*A_{k,3}^{d_{3}}*A_{k,4}^{d_{4}}.$$ (2)

The predicted viability of a four-drug combination, *v*_*c*_ can then be obtained using a modified form of Eq (1) as

$$v_{c}=e^{\beta_{0}}*{\left(A_{1,c}\right)}^{\beta_{1}}*\ldots *{\left(A_{p,c}\right)}^{\beta_{p}},$$ (3)

where the coefficients *β*_*k*_ are determined using the linear regression on the single-drug data.

## Results

### Hypoxia-induced myoblast death *in vivo*

**Fig 1:**
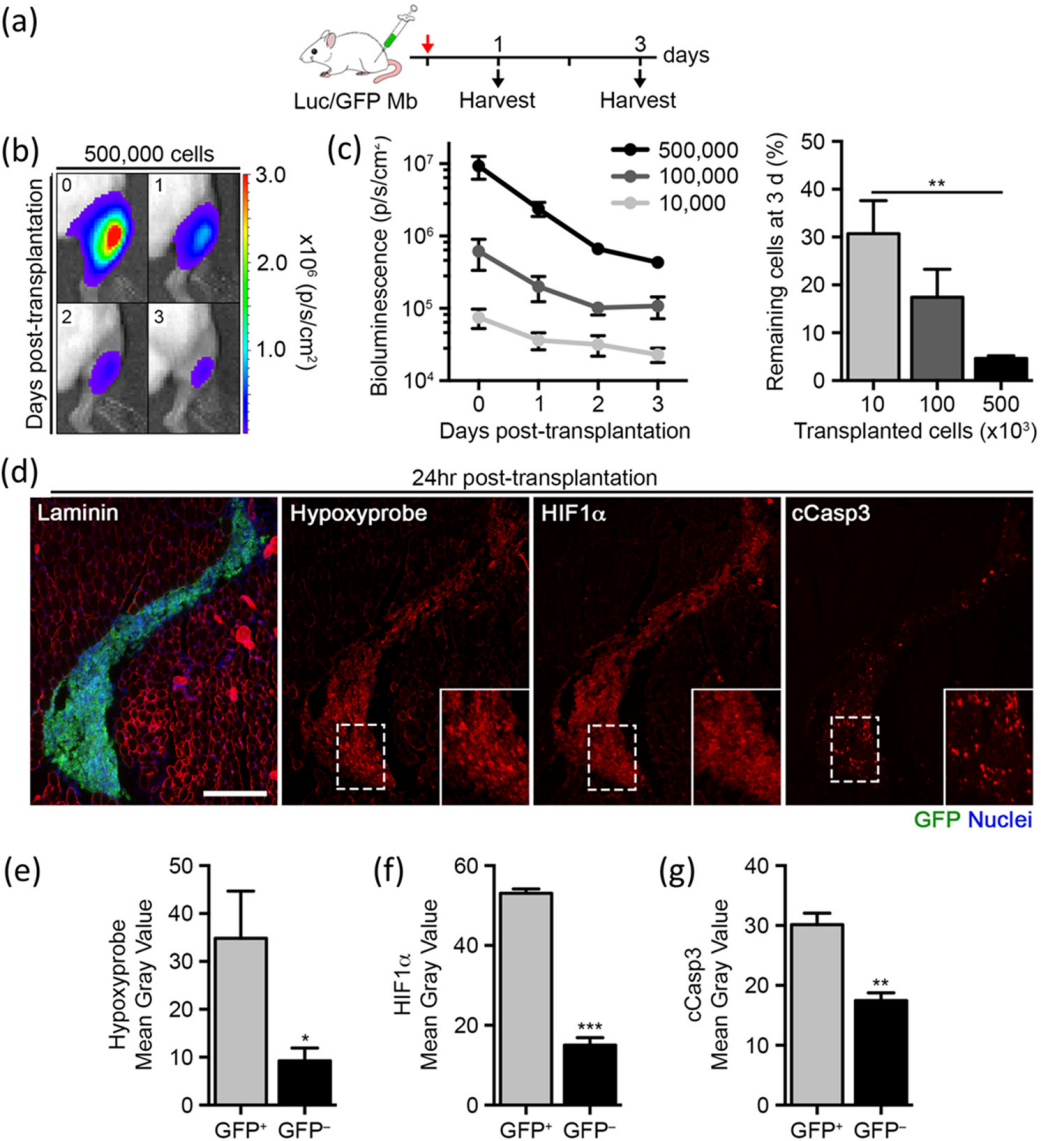
Hypoxia-induced cell death in transplanted primary myoblasts. (a) Schematic representation of myoblasts isolated from Luciferase x EGFP mice and transplanted into the tibialis anterior muscle of NOD/SCID recipient mice. (b) Representative images of bioluminescence signaling in recipient mice at the indicated time points post-transplantation (values indicated as photons s^–1^ cm^–2^). (c) Quantification of bioluminescence signaling following the transplantation of 500,000, 100,000 or 10,000 cells over 3 d (n = 4, left). Quantification of the percentage of surviving myoblasts 3 d post-transplantation (right). (d) Representative images of GFP+ myoblasts 1 d post-transplantation (green = GFP, blue = nuclei and red = laminin, hypoxyprobe, HIF-1α or cCasp3 as indicated). Scale bar, 200 μm. (e-g) Quantification of mean gray values from hypoxyprobe, HIF-1α or cCasp3 staining in areas occupied by GFP^+^ donor myoblasts (or GFP^−^control) (n = 4). Data are represented as average ± SEM. Student’s t test was used for all statistical analyses (*P < 0.05, **P < 0.01, ***P < 0.001).

Myoblasts tend to remain localized at the injection site following their intramuscular transplantation, and the majority of these myoblasts die shortly after delivery [8–11, 31, 32]. To determine the relationship between donor cell concentration and survival rate, we derived primary myoblast lines from *Luciferase x EGFP* double transgenic mice. Increasing cell numbers were injected into the tibialis anterior (TA) muscles of hind limb irradiated immunodeficient NOD/SCID mice and harvested after 1–3 days (Fig 1A). We took advantage of noninvasive bioluminescence imaging (BLI) to dynamically monitor cell number after transplantation [8, 33]. This strategy takes advantage of the luciferase protein, which in the presence of its specific substrate, luciferin, emits light that can be detected by a CCD camera [21]. As expected, donor myoblast number was significantly reduced after 3 days in all recipient muscles (Fig 1B). Notably, myoblast survival was inversely correlated to the number of cells transplanted, as only 4.6 ± 0.6% remained after 3 days when transplanting 500,000 cells as opposed to a 30.7 ± 6.9% survival rate when transplanting 10,000 cells (Fig 1C). These results confirm the high rate of donor cell loss following intramuscular transplantation and suggest that myoblast survival cannot be efficiently overcome simply by increasing the number of cells transplanted.

To evaluate the role played by hypoxia on myoblast survival, hypoxyprobe (pimonidazole hydrochloride) was administered intraperitoneally 1 day post-transplantation. This small molecule selectively binds to oxygen-starved cells and can be detected by antibody recognition through histological assessment [11]. Indeed, donor myoblasts formed a cellular bolus within the tissue strongly positive for hypoxyprobe, indicating hypoxic conditions specifically in the transplanted area (Fig 1D and 1E). We also detected the upregulation of hypoxia inducible factor 1 alpha (HIF-1α), an early mediator of the cellular response to low oxygen conditions, in the transplanted region (Fig 1D and 1G). Finally, a significant amount of apoptosis was identified by cleaved caspase-3 (cCasp3) expression (Fig 1D and 1G). Together, these data associate poor myoblast survival with hypoxic conditions in recipient tissues.

### Screening of kinase inhibitors

**Fig 2:**
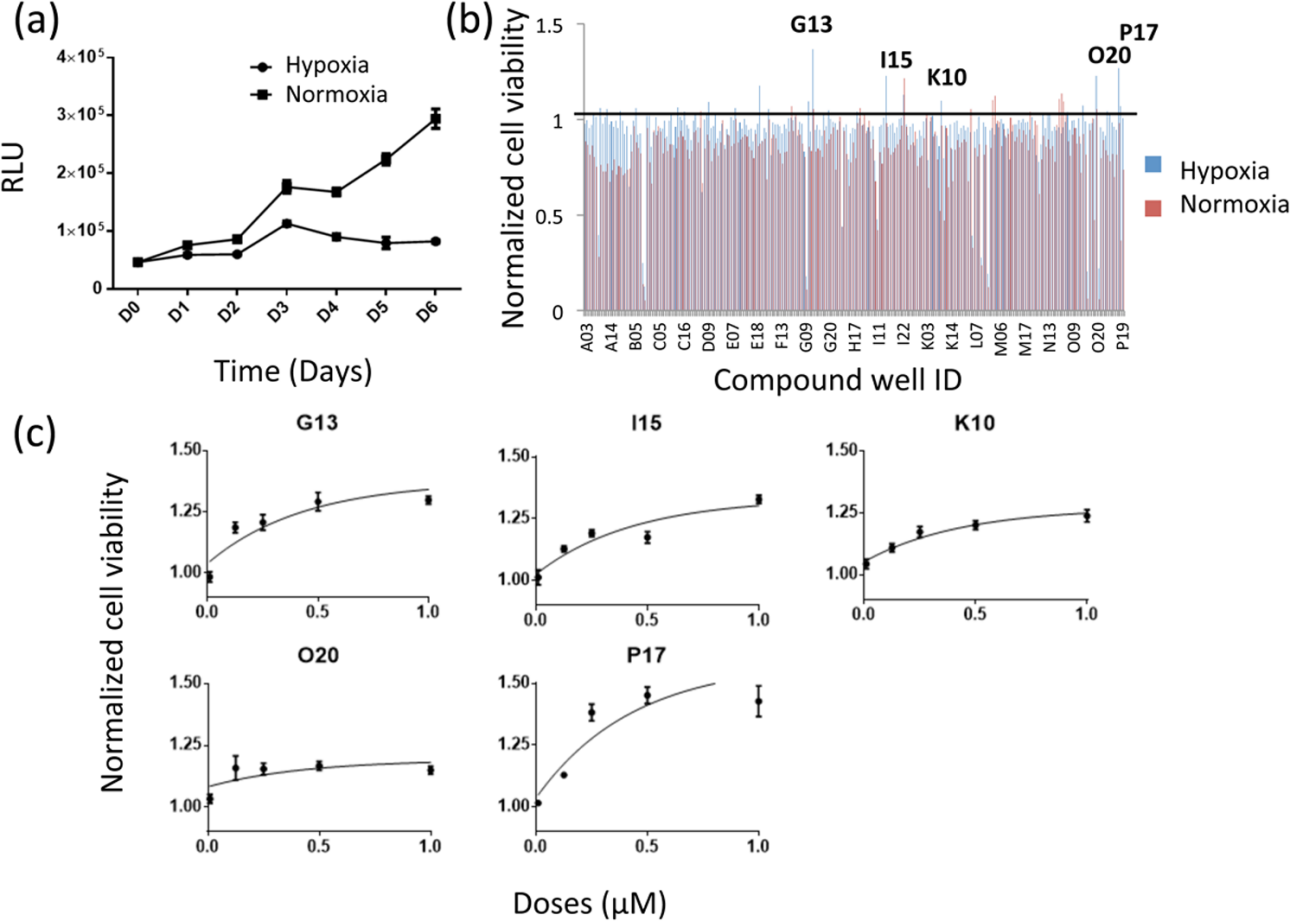
Primary screening of kinase inhibitor libraries. (a) Myoblast proliferation was observed under hypoxic and normoxic conditions for 6 days. Cellular ATP production was measured by an ATPlite assay and is indicated in Relative Luminescence Units (RLU) (n = 12, p<0.01). (b) The EMD kinase inhibitor library was screened for their capability to protect cells from 0.1% hypoxia at 1 μM concentration. Five kinase inhibitors were selected based on their efficiency in improving cell viability in hypoxic condition. (c) Dose-dependent protective effects of five candidate compounds on primary myoblasts cultured in hypoxic condition for 5 days. The cell viability in the compound-treated groups was normalised against DMSO-treated control group using ATPlite assay (n = 6, p<0.01 for all groups).

To develop a phenotypic screen for compounds able to impact myoblast growth and survival, we cultured primary myoblasts in normoxic (20% O_2_) or hypoxic (0.1% O_2_) conditions for five days. As expected, the number of primary myoblasts was significantly reduced in hypoxic as compared to normoxic culture conditions (Fig 2A). We then screened the EMD kinase inhibitor library for their ability to overcome hypoxia-induced growth retardation and cell death at 1 μM. Among 244 kinase inhibitors screened, five kinase inhibitors were shown to significantly improve primary myoblast viability by at least 10%. These effects were confirmed with follow-up dose response experiments and proportional increase in cell survival was observed with increase doses of kinase inhibitors. (S1 Table and Fig 2B and 2C). These kinase inhibitors are G13, P17, O20, I15, and K10. These kinase inhibitors did not show any effects in normoxic condition. The name and targets of these kinase inhibitors are described in Table 1.

**Table 1:** Description of the drug targets.

Well ID	CAS number	Inhibitor name	Main inhibitor target
G13	856436-16-3	JAK3 Inhibitor VI	JAK3
I15	608512-97-6	PKR Inhibitor	PKR
K10	142273-20-9	Kenpaullone	GSK-3b2, CDK1/B2, CDK22/A2, CDK5/p25, CDK2/E
O20	666837-93-0	SU9516	CDK2/A1, CDK1/B1, CDK4/D11
P17	1177150-89-8	WEE1/CHK1 Inhibitor	WEE1, CHK1

### Fully factorial analysis of the selective kinase inhibitors

**Fig 3:**
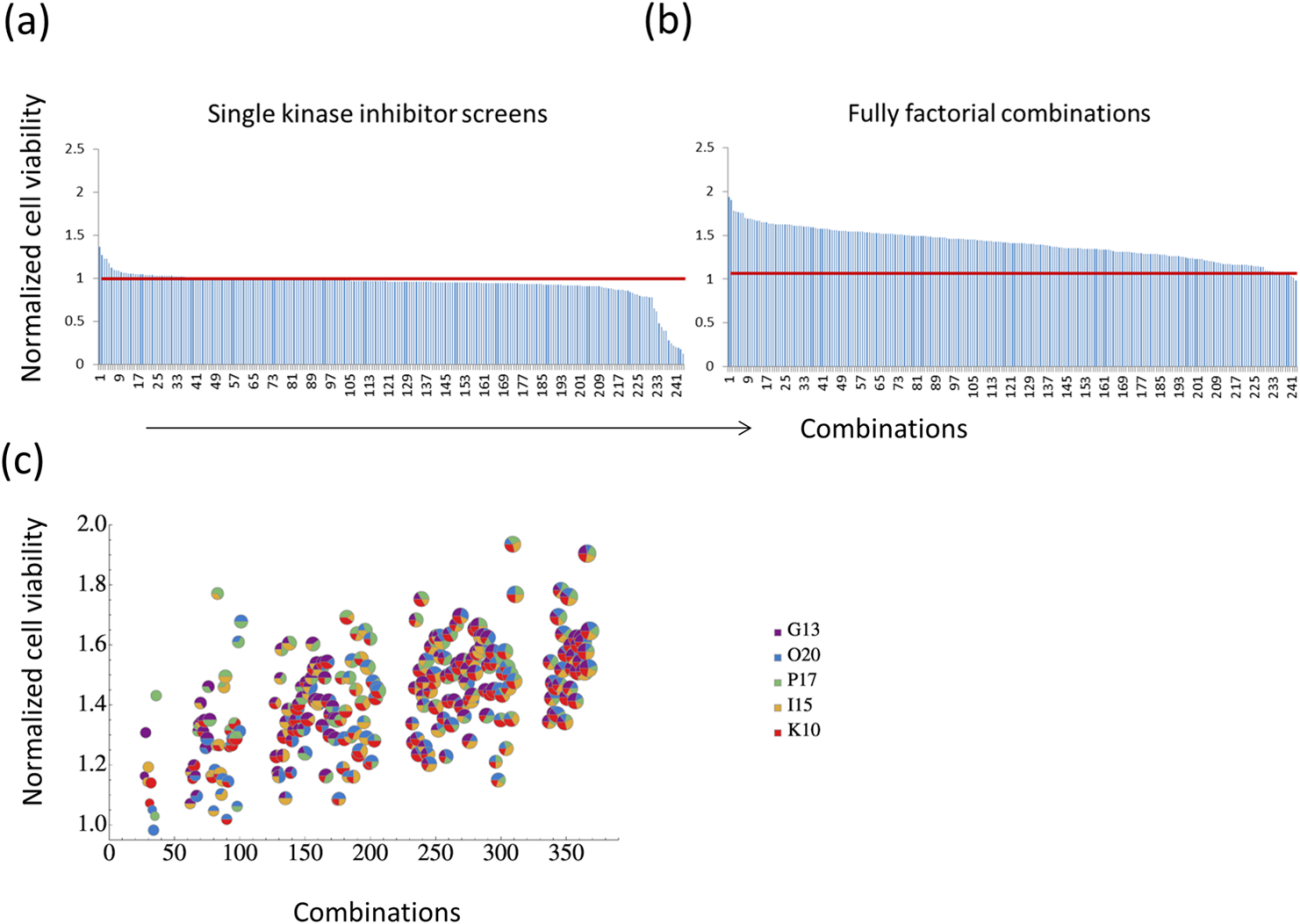
Fully factorial combination studies. (a) The EMD kinase inhibitors were rank ordered according to their ability to protect myoblasts in hypoxia (n = 4) (b) All possible combinations of five candidate compounds were tested at two different doses (1 μM as high dose and 0.25 μM as low dose) for their ability to protect myoblasts in hypoxia. The cell viability in the compound-treated groups was measured relative to the DMSO-treated control group at 5 days of culture using ATPlite assay (n = 3). The combinations were rank ordered according to their protective capability. (c) The normalised cell viability for each combination was shown. The pie symbols show the relative contribution of five kinase inhibitors.

For the fully factorial experiments, two dose levels of each compound were chosen for the combinations: a low dose at 0.25 μM and high dose at 1 μM. This resulted in 243 different combinations. As individual compounds, G13 and P17 showed the highest efficacy in improving cell survival under hypoxia (S2 Table). Cell viability was markedly improved by some of the combinations in comparison to that achieved by single agents (Fig 3A and 3B). The highest increase in cell viability was achieved by a combination of O20, P17, I15, and K10, which showed a 93.5% increase in cell viability compared to controls (S2 Table). Fig 3C clearly demonstrates that combinations including a larger number of drugs promote cell viability more efficiently. The synergy analysis provides a measure of the relative strength of synergistic or antagonistic effects in different combinations. The synergy/antagonism was quantified using three different measures: the highest single agent (HSA) excess, the highest pair agents (HPA) excess and the Bliss excess (see Methods for their mathematical definitions). When pairs of the drugs were analyzed, P17 showed significant synergy with O20 and I15. In addition, P17 was synergistic with most of other drugs used for combinations whereas G13, the most efficient inhibitor, did not show synergies with other drugs except for P17 (S3 Table). The strongest synergies were achieved by some of the larger combinations, mostly combinations between four drugs, indicating that a combinatorial approach is an efficient strategy to inhibit hypoxia-induce growth arrest/cell death.

Secondary confirmatory assays were also performed in addition to ATPlite to ensure that the measurement of ATP contents is reflective of viable cell numbers. ATP contents measured by ATPlite assay was well correlated with viable cell number counted by Trypan blue exclusion assay with R^2^ = 0.9901 (S1 Fig). This was also confirmed with fluorescence-based cell counts and activated caspase-3 staining for the selected treatment groups. The pattern of cell survival by both cell viability measurements is in good agreement with activated caspase3 staining (S1 Fig).

### Pathway and regression analysis for the key kinases/synergy predictions

**Fig 4:**
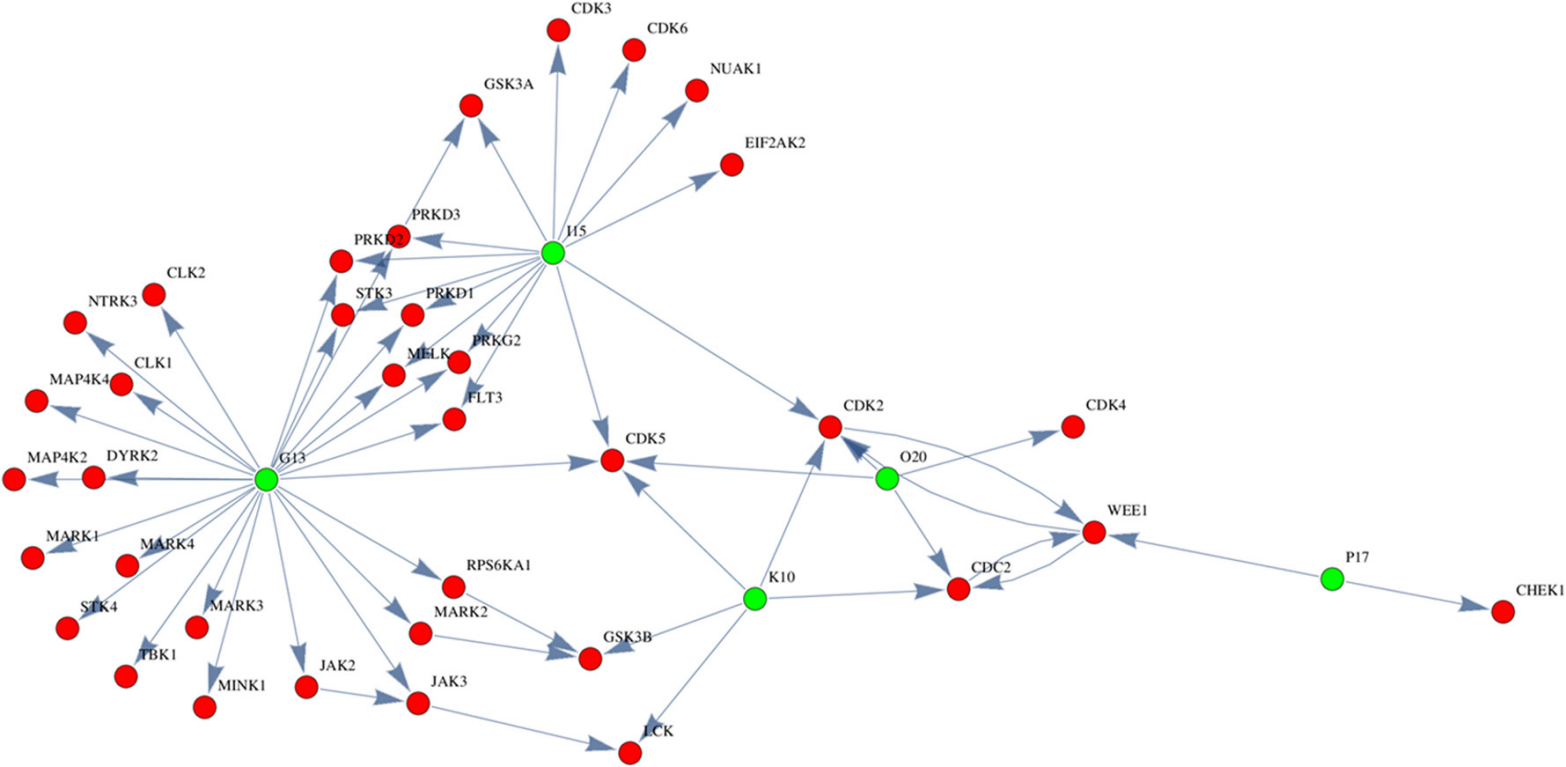
Pathway analysis. Green nodes represent five selected kinase inhibitors whereas red nodes represent direct/indirect targets of those inhibitors. Each edge represents the influence of the inhibitors or upstream kinases on the phosphorylation levels of downstream kinases.

To understand the pathways affected by the kinase inhibitors, we analyzed networks of kinase inhibitor and their targets. Fig 4 shows the subnetwork consisting of the five top inhibitors, their first neighbors, and corresponding interactions (see Methods). Each node represents kinases that are targeted by the inhibitors and each edge represents the influence of the inhibitors or upstream kinases on the phosphorylation levels of downstream kinases. The analysis shows that inhibitors I15, K10 and O20 act on targets that are highly enriched in cell cycle related kinases. Among those targets that have multiple edges are CDK5, CDK2, CDC2, WEE1, and GSK3β. In particular, CDK5 is a direct target of four candidate inhibitors. It is plausible that unshared parallel pathways should be targeted simultaneously to trigger synergistic protective mechanism. For example, I15 and G13 share multiple common targets but did not show any synergies whereas P17 was synergistic with I15 and O20, without any direct sharing of targets (Fig 4 and S3 Table).

**Fig 5:**
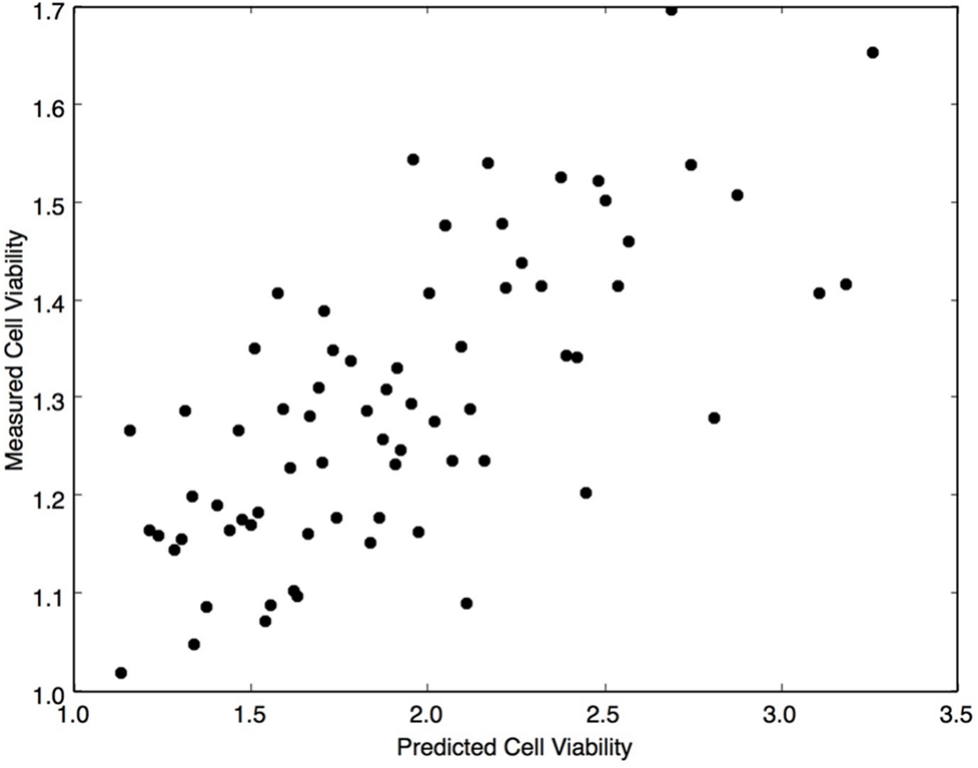
Prediction of combination effects. The correlation between KIEN combinatory predictions and experimental results are shown. Each dot corresponds to the myoblast viability obtained from 72 combinations between four drugs: G13, O20, I15, and K10 (R^2^ = 0.70, P<0.00001).

We also built a regression model based on the kinase inhibitor screening results and a published dataset containing target profile of the kinase inhibitors (290 targets) using the KIEN method, which we have recently developed [30]. A limitation of this approach was that full profiling information for one of our kinase inhibitors (P17) was not available in the published dataset, thus the analysis was performed only with G13, I15, K10, and O20. The coefficient *β*_*k*_, a measure of the protective impact of each kinase, was calculated and the kinases were ranked using this parameter in order to estimate the main kinases influencing the protective mechanisms against hypoxia (see Methods). The coefficients *β*_*k*_ for CDKs such as CDK1, CDK2 and CDK5 ranked at the top (Table 2 and S4 Table), confirming our pathway analysis. In addition, CDKs also ranked high when we analysed our previous screening data obtained in a neuronal cell line [34], suggesting that CDKs play important roles in protection against hypoxia [34] in multiple cell types (Table 2 and S4 Table). This comparison also shows that cell-specific protective kinases can also be identified. The effects of combinations of the mentioned four kinase inhibitors could also be predicted using a modification of KIEN. Fig 5 shows predictions obtained with KIEN and the corresponding experimental results. Each of the 72 points indicates a combination of 4 drugs (G13, O20, I15, and K10) with different dosages. The Pearson correlation between the predicted and measured values is 0.70 (P<0.00001). In summary, pathway and target analysis revealed CDKs as the main target kinases with CDK5 being in the center of target kinase networks.

**Table 2:** Top 10 kinases that show a protective response in hypoxia when inhibited.

Kinases (Myoblast)	*β*_*k*_ Coefficient	Kinases (HT22)	*β*_*k*_ Coefficient
**CDK5**	-0.0125	**CDK5**	-0.028
**MYLK2**	-0.0124	**STK4**	-0.0209
CDK1	-0.0099	FLT3	-0.0177
CLK2	-0.009	MKNK2	-0.0168
**STK4**	-0.0074	ZAK	-0.0164
MAP4K4	-0.0068	**MYLK2**	-0.0157
MARK4	-0.0065	CDK6	-0.0153
CDK2	-0.0059	LIMK1	-0.0149
STK3	-0.0052	MAP3K2	-0.0111
MELK	-0.0032	KIT	-0.0108

The data were obtained using KIEN on the entire kinase inhibitor library. The coefficient *β*_*k*_, a measure of the protective impact of each kinase, was calculated and the kinases were ranked using this parameter in order to estimate the main kinases influencing the protective mechanisms against hypoxia. In bold we show kinases important for the response of both cell lines.

## Discussion

Several studies have demonstrated that the majority of donor myoblasts (70–90%) die in the first few days upon intramuscular transplantation and this in part correlated with a local increase in hypoxia in the transplanted area [8, 9, 11, 32]. Evidence in the literature indicates that hypoxia is a major hurdle for cell implantation in skeletal muscles [11, 12]. It has been shown that the donor myoblasts form cell pockets of hypoxic zones after transplantation. This phenomenon is also confirmed in our results showing that the region of myoblast transplantation develops a strong hypoxic condition with HIF1α upregulation at the core. The transplanted myoblasts went through apoptosis with significant activation of caspase-3, associating hypoxia with cell death pathways in transplanted myoblasts. The identification of combinations of small molecules that overcome hypoxia-associated cell death might benefit the development of transplantation strategies for patients with skeletal muscle degenerative diseases.

In this study, the exposure of myoblasts to 0.1% hypoxia for five days was devised as *in vitro* condition to induce hypoxia-induced cell death/growth arrest. While the culture of myoblasts *in vitro* may result in adapted nutrient availability and metabolism, these cells are nonetheless able to proliferate more rapidly under normoxic rather than hypoxic conditions (Fig 2A). Another aspect to consider is that cells might adapt to anaerobic metabolism during hypoxia, resulting in glycolysis and lactic acidosis [35]. However, altered metabolisms are considered as a part of the hypoxic phenomena. Further studies are needed to determine what aspects of hypoxia-induced cellular damage our candidate factors specifically protect the cells from, including but not limited to metabolic perturbations.

Herein, five potent kinase inhibitors that protect myoblasts from hypoxia-induced death/growth arrest were identified. Among the intracellular targets, cell cycle kinases (CDKs) were shown to play key roles and growing evidence suggests that they are linked with hypoxia-induced cell death[36–38]. Upon hypoxia exposure, cells activate the DNA Damage Response (DDR), mediated by ATM, ATR, CHK1 and CHK2 [39, 40], key controllers of cell cycle checkpoint pathways [41]. One of the main regulators of CDKs expression, Notch, has also been reported to play an important role in the response to hypoxia [42, 43] and is a critical regulator of cell fate decisions in muscle progenitors [44, 45]. Additionally it has been shown that upon hypoxia another cell cycle regulatory kinase, WEE1, a target of the most efficient inhibitor P17, is rapidly activated [46]. WEE1 is a tyrosine kinase that phosphorylates cdc25 at the G2/M checkpoint and prevents the progression to mitosis [47]. The targets of G13, the second most effective inhibitor, are JAK2 and JAK3, which have been reported to influence CDKs and Glycogen synthase kinase-3 beta (GSK3β) [48, 49]. The JAK/STAT pathway is known to play a role in multiple cellular stress conditions, including hypoxia, and pharmacological inhibition of both JAK2 and STAT3 was recently shown to promote satellite cell expansion during the early stages of muscle repair [50–52]. Interestingly, it has been shown that GSK3β antagonizes both HIF-1α protein accumulation and the autophagic pathway in hypoxia conditions and promotes apoptosis through activation of caspase-3 and release of cytochrome c from the mitochondria [15, 53]. Indeed, efficient GSK3 inhibitors have been shown to promote cell survival in several stress conditions [54].

Our fully factorial studies verified the effectiveness of combinatorial approaches while pathway analysis revealed a few important pathways associated with hypoxia-induced myoblast death. In particular, our study shows that CDK5 is at the center of the target network. The KIEN regression model also predicted CDK5 as a key target kinase that has protective impact on the cells in hypoxia when the resulting coefficients were ranked. Interestingly, KIEN analysis also ranked CDK5 highly when performed for the HT22 neuronal cell line, suggesting that CDK inhibition might be a general protective mechanism relevant to hypoxia in more than one tissue. The important role of CDK5 in hypoxia-associated cell death has previously been described, supporting our findings [36, 55]. Albeit in non-myoblast cells, CDK5 was reported to control ischemic/hypoxic damage and mediate excitotoxic neuronal cell death [36]. For example, viral-mediated dominant negative CDK5 expression was shown to inhibit death induced by hypoxia in a mouse stroke model [36]. In addition, the protective efficacy of CDK and GSK3 inhibitor was reported in hypoxia-ischemia mouse model [38]. CDK and GSK3 are two important targets linking the candidate inhibitors from our analysis. Taken together, these literatures support the validity of our regression model that identified CDK5 as a key kinase from the kinase inhibitor library screening. In the future, we could simultaneously target the highly ranked kinases in combination with CDK5 in an effort to achieve improved protection against hypoxia.

Finally, our results support the advancement of combinatorial approaches, rather than single drug methods, to the treatment of hypoxia-induced cell death. These interventions are likely much closer to the combinatorial control we find in nature, (for example by transcription factors or microRNAs) but until now, not in pharmacology. We report both additive and synergistic benefits gained by the use of kinase inhibitors to improve cell viability, increasing their efficiency in this context. While the development of precisely regulated controls will be important to solve concerns of increased risk of toxicity with multiple agents, well-modulated combinatorial drug regimens may rather overcome drug side effects by minimizing biological compensations and reducing administered doses of single agents [56]. Therefore, the search for optimal combinations of small molecules may not only benefit the development of treatments to improve existing therapies but also preventing any toxicity associated with single drug regimens.

While potentially more efficient once identified, the validation of combinatorial approaches via fully factorial screenings of even modest drug libraries may prove costly and require extremely large throughput. To alleviate these concerns, our results demonstrate that KIEN [20] can also predict which combinations of kinase inhibitors will be effective for hypoxia protection given information from the screen of a library of single drugs. This method may prove to be a useful tool to predict and select kinase inhibitor combinations from large libraries of characterized kinase inhibitors available for post-hoc analysis, reducing the resources required for experimental validation. The coefficients of the model (Table 2 and S4 Table, including the complete list of kinases) indicate the optimal amount of inhibition of each target kinase responsible for the protective effect, for a large number of kinases. The potential of the KIEN results is therefore not simply to provide a list of targets to be completely inhibited but include the appropriate quantitative amount of inhibition, further minimizing drug toxicity concerns.

In summary, this study successfully identified kinase inhibitors that overcome myoblast cell death/growth arrest induced by hypoxia. Through fully factorial studies, we found synergistic combinations that significantly improve myoblast survival in hypoxia. In addition, pathway and regression analysis identified the key kinases involved in the hypoxia-associated reduction of viability in myoblasts. Future studies might investigate the in vivo effects of these compounds.

## Supporting Information

S1 Fig (a) The standard curve between ATPlite contents and viable cell number measured by Trypan Blue exclusion assay. (b) The comparison of relative cell survivals measured by ATPlite assay and flurescence-based automatic cell counts in primary myoblasts untreated or treated with the indicated inhibitors (n = 4). (c) Detection of activated caspase-3 in primary myoblasts untreated or treated with the indicated inhibitors. (blue = nuclei and red = cCasp3), Scale bar = 50 μM
(TIF)

S1 Table The kinase inhibitors were tested in myoblasts cultured in hypoxic condition and rank-ordered according to the normalized cell viability. (XLS)

S2 Table Fully factorial analysis of five candidate compounds. H: high dose (1uM) L: low dose (0.25uM)
(XLS)

S3 Table Drug synergy was calculated based on three different methods: excess with respect to the Bliss independence model (Bliss excess), excess with respect to the highest single agent (HPA) and excess with respect to the highest pair agents (HPA). (XLSX)

S4 Table The coefficients of the model indicate the optimal amount of inhibition of each target kinase responsible for the protective effect in myoblast. (XLSX)
