# Supplementary figures and images for: Combinations of Kinase Inhibitors Protecting Myoblasts against Hypoxia
Source: PLoS One. 2015 Jun 4;10(6):e0126718. doi: 10.1371/journal.pone.0126718 (PMC4456388; doi:10.1371/journal.pone.0126718)

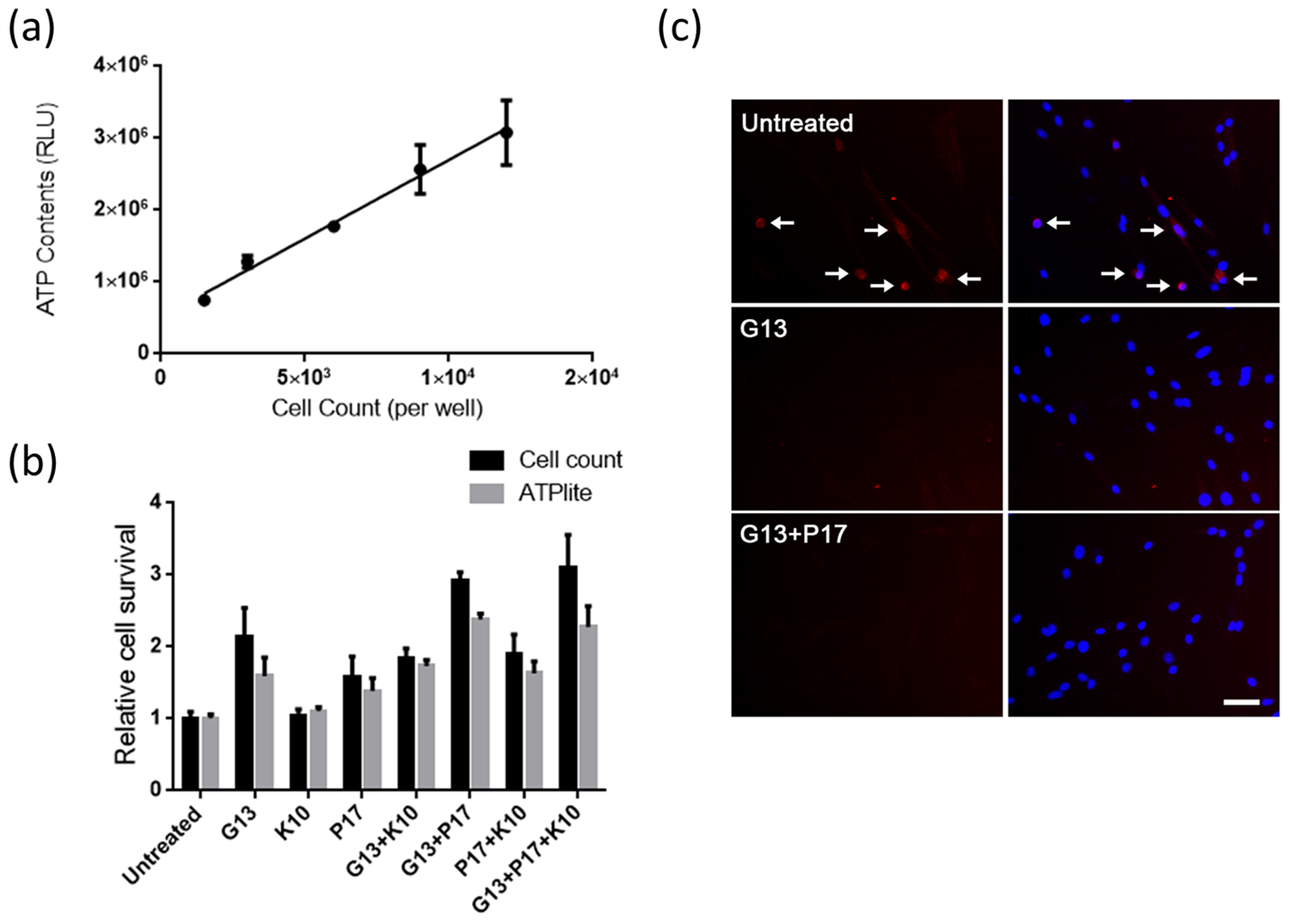

Supplement: S1 Fig — (a) The standard curve between ATPlite contents and viable cell number measured by Trypan Blue exclusion assay. (b) The comparison of relative cell survivals measured by ATPlite assay and flurescence-based automatic cell counts in primary myoblasts untreated or treated with the indicated inhibitors (n = 4). (c) Detection of activated caspase-3 in primary myoblasts untreated or treated with the indicated inhibitors. (blue = nuclei and red = cCasp3), Scale bar = 50 μM (TIF) [file pone.0126718.s001.tif]
